# The precarization of the Mexican nursing labor market: a repeated cross-sectional analysis for the period 2005–2018

**DOI:** 10.1186/s12960-019-0417-x

**Published:** 2019-11-21

**Authors:** Patricia Aristizabal, Gustavo Nigenda, Edson Serván-Mori

**Affiliations:** 1 Iztacala Faculty of Higher Studies, National Autonomous University of Mexico, Mexico City, Mexico; 2 National School of Nursing and Obstetrics, National Autonomous University of Mexico, Mexico City, Mexico; 3 Center for Health Systems and Research, National Institute of Public Health, Cuernavaca, Mexico

## Abstract

**Background:**

Precarization of labor conditions has been expanding over the last three decades as a consequence of global economic transformations. The health workforce labor market is exposed to these transformations as well. In Mexico, analyses of the nursing labor market have documented high levels of unemployment and underemployment; however, precarization has been not considered as a relevant indicator in these analyses. In this study, precarization is analyzed using a quantitative approach to show its prevalence and geographic distribution between 2005 and 2018.

**Methods:**

A repeated cross-sectional study was carried out with data from the population-based National Occupation and Employment Survey (ENOE in Spanish) which is administered quarterly to people 15 years or older in over 120 000 households. All individuals who reported having nursing training (technical or university level) were selected for this study. Our main variable was labor precariousness, which included three dimensions: (i) economic, (ii) regulatory, and (iii) occupational safety. We show the evolution of the relative weight of nursing activity between the years 2005 and 2018, the main socio-demographic characteristics of nurses as well as their main labor conditions, and the geographic distribution of precariousness for the 32 federal states in México.

**Results:**

Four of the five indicators of labor precariousness increased among the group of nurses analyzed: (a) the percentage of people with no written contract, (b) the percentage of people with incomes lower than two times the minimum wage, (c) the percentage of nurses without social security, and (d) the percentage of nurses without social benefits. The percentage of nurses that work under some condition of work precariousness increased from 46% in 2005–2006 to 54% in 2018. Finally, the number of states with high precariousness level increased from seven in 2005–2006 to 17 in 2018.

**Conclusions:**

Throughout Mexico, nursing precariousness has expanded reaching 53% by 2018. The advancement of precarization of nursing jobs implies a reduction in the capacity of the Mexican health system to reach its coverage and care goals as nurses represent 52% of all available workers that provide direct services to the population.

## Background

Universal health coverage (UHC) is an issue on the global agenda to which many countries are committed [1, 2]. Its achievement is considered a fundamental strategic goal to guarantee population access to health services. In 2012 in Mexico, it was stated that the country had achieved Universal Health Coverage through a strategy that focused financial, material, and human resources on populations that had historically lacked insurance and attention [3]. In 2003, Seguro Popular de Salud (SPS), or Popular Health Insurance, was created to guarantee the provision of these resources to populations without traditional social security coverage. Social security coverage and SPS coverage converged to achieve universal coverage.

However, the declaration of universal coverage by itself does not guarantee that the services provided by the institutions will have positive effects on health since this requires the provision of a further set of resources, particularly human resources, distributed equitably and according to population needs, in health centers and hospitals within the system. Prior to the UHC declaration in 2012, the Mexican health system had achieved a significant increase in the availability of human resources including nurses. In fact, using SPS financial resources, by 2008, more than 55 000, mainly doctors and nurses, were hired to provide services in public health care units [4, 5]. However, availability was still far from the human resources of other countries with similar incomes, and Mexico had serious geographical distribution problems as well.

Thus, between 2000 and 2015, the Mexican Health System has significantly increased the availability of nurses at all levels, which grew from 1.2 to 2.6 nurses per 1000 inhabitants [6]. However, this exponential growth masks enormous geographical disparities in nurse availability. While in Mexico City there were 5.2 nurses per 1000 inhabitants, poor states such as Chiapas and Oaxaca had only 2.2 and 2.1 nurses per 1000 inhabitants respectively [7].

These gaps in availability and distribution show only some dimensions of the distortion in the distribution of nursing resources in Mexico [8]. The labor market perspective is important in understanding how this availability needs to be considered as an initial stage in linking nursing personnel in the health system, and labor conditions as a subsequent stage in order to promote their effective participation in the production of services [9, 10].

In Mexico, there are few studies that address changes in the labor market with a precarization framework [11, 12]; however, growing phenomena associated with precarization are being recognized, such as the flexibility of the labor market, the loss of social benefits, and the increase in informality, all of which demonstrate the deterioration of labor relations and violate the rights of employees [13, 14].

Mexican labor market precarization has been growing over the past 20 years. According to Garcia [15], temporary hiring, absence of contracts, and lack of access to health services as a labor right are aspects that characterize the precariousness of the Mexican labor market. Also, García states that the proportion of precarious jobs decreases with an increase in the educational level of the worker and that government jobs have a lower percentage of precarization in relation to other economic sectors.

This trend may be reflected by health worker labor markets. Regarding nurses, the country has to respond not only to the need to increase their availability, but also to regulate a labor market that should be able to offer greater opportunities for linking nurses to areas that correspond to their training and according to national and international labor rights. Appropriate regulation would help to balance the supply, demand, labor insertion, and distribution of nursing personnel [16] and consequently to reduce harmful phenomena such as precarization.

As an initial approach to the study of the precarization of the nursing labor market in Mexico, we present original estimates about the degree of job precarization faced by Mexican nurses, their evolution, and each of their dimensions (economic, regulatory, and labor security). In particular, this study aims to describe temporal and geographic patterns of precarization in Mexico between 2005 and 2018 and to discuss initial implications for the Mexican Health System.

## Methods

### Data

A repeated cross-sectional study was carried out between 2005 and 2018, with data from the National Occupation and Employment Survey (ENOE in Spanish). The ENOE is administered quarterly to people 15 years or older in over 120 000 households. It is the main reference for obtaining high-quality statistical information for labor characteristics of the economically active and inactive Mexican population, as well as other individual and household demographic and economic variables.

The ENOE is designed under a rotating panel with cycles of five visits, so that each quarter 20% of the original sample is replaced. This survey is administered by the National Institute of Geography and Information Statistics (INEGI in Spanish) and is available on their website (https://www.inegi.org.mx/programas/enoe/15ymas/). The ENOE sampling scheme is a probability, two-phased, stratified, clustered sample; its ultimate sampling units are private households (non-institutions), and its observation units are persons in selected households. It accounts for a maximum non-response rate of 15% and can generate results representative of national, state and rural, urban, and metropolitan Mexican populations.

The study population included individuals surveyed who reported having nursing training (technical or university levels) employed or not, men and women. For the purposes of this research, all households that were visited for the first time in each quarter and year were selected. This allowed the elimination of quarterly seasonality from the behavior of the analyzed data, capturing the greater heterogeneity in the data and reducing the redundancy of observation units (households and population of interest).

Thus, the initial study sample included all health professionals (physicians, licensed or unlicensed nurses, and other occupational categories with technical or licensed training) from 15 to 70 years of age (*n* = 73 379, in all years, *N* = 18 214 684). Of this group, nurses accounted for 30 250 (*N* = 7 739 074). After excluding from analysis people without complete information in all of the characteristics of interest (7.9%), the final sample analyzed was comprised of 27 942 nurses (*N* = 7 123 763).

### Variables

Our main variable was work precariousness. In this study, work precariousness was measured through three dimensions proposed by Román-Sanchez, [17]: (i) economic, which considers the level of income received in exchange for labor force, using minimum wage as a reference; (ii) regulations, which include the contract and duration of the working day, specifically, whether or not there is a written contract; and (iii) occupational safety, which includes the affiliation to social security and social benefits, measured by receiving services in health institutions and having at least one social benefit. Given its definition, this variable could only be measured in the 15 074 subjects (*N* = 3 699 282) who reported being economically active, employed, and subordinated and remunerated.

Operationally, a non-weighted score of additive work precariousness was built and based on the sum of five dichotomous variables (yes = 1/no = 0) that included the three dimensions mentioned above: (i) salary, equal to 1 if the individual reported up to two times the minimum wage; (ii) workday, equal to 1 if the individual works less than 34 h and more than 48 h (part-time or excessive); (iii) contract, equal to 1 if the individual reported not having a written contract; (iv) social benefits, equal to 1 if the individual does not have social benefits; and (v) social security, equal to 1 if the individual reported not having access to services in health institutions. All these variables were counted as zero in the event that they did not comply with the aforementioned condition. Thus, the calculated score takes values from 0 to 5, which refer to the minimum or maximum possible work precariousness. We classified the study participants into four levels of precariousness: without precariousness (score equal to 0 or without any lack of work), low precariousness (score equal to 1), medium precariousness (score equal to 2 or 3), and high precariousness (score equal to 4 or 5).

Other analyzed variables included socio-demographic and labor characteristics such as having a bachelor’s degree or a university degree, sex (female = 1, male = 0), years of age (24 or less, 25 to 44, 45 to 64, and 65 or more), marital status (married or unmarried, single, separated, divorced, or widowed), being employed in the health sector or another sector, being employed in the public or private economic sector, the number of jobs (one or two or more), and the locality of residence (rural, semi-urban, urban, and metropolitan).

### Analysis

First, we describe the evolution of the relative weight (% and CI95%) of nursing activity within total activity and/or that of health-related professions from 2005 to 2018.

The main socio-demographic characteristics of the nurses were also described, as well as the main labor characteristics of the study population: economic activity, employment, subordination and remuneration, number of jobs, the economic sector in which participants worked, and the level of each of the job precariousness variables (no, lowest, middle, and highest work precariousness). In order to make the analysis coincide with the last three federal governments, this description was used for the periods between 2005 and 2006 (President Fox’s government), 2007–2009 and 2010–2012 (President Calderon’s government), and 2013–2005 and 2016–2018 (President Peña’s government).

Finally, we show the geographical distribution in quartiles of precariousness for the 32 federal states in México. Cutting thresholds are based on the period between 2005 and 2006.

We report percentages and CI95%. Differences were evaluated throughout the period analyzed by calculating the *P* for trend. These analyses considered the design effect of the survey. Analysis was performed using the statistical package Stata MP v15.1.

## Results

**Fig. 1:**
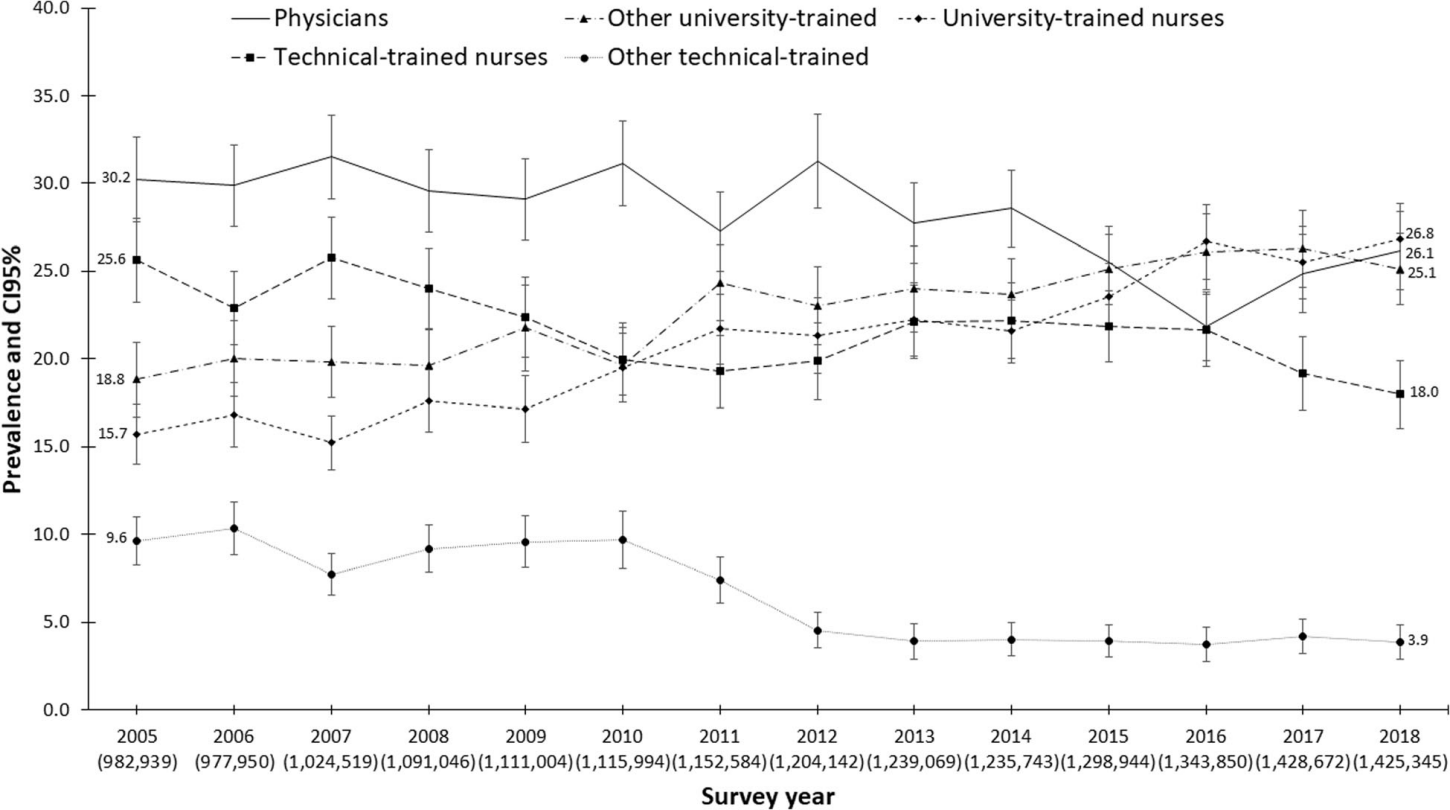
Distribution of health occupations in Mexico, 2005–2018. Estimations considered the design effect of the survey. In parenthesis weighted population. Data source: National Occupation and Employment Survey (ENOE in Spanish) 2005–2018

According to the data analyzed and throughout the study period, four out of every ten individuals of the analyzed population with training in the health field reported studying nursing (technical or university level training) and three out of ten studied medicine (Fig. 1). However, among the total population of health workers, the participation of university-trained nurses showed the highest growth, practically doubling in 14 years, from 15.7% in 2005 to 26.8% in 2018.

Regarding the socio-demographic profile of subjects with nursing training, four results stood out: (i) the percentage of men went from 6% between 2005 and 2016 to 15% in between 2016 and 2018 (*P* < 0.001); (ii) the percentage of people 24 years or less grew almost 32% (*P* < 0.001), while the group of 25- to 44-year-olds decreased by 21% (*P* < 0.001); (iii) university-trained nurses increased by 43% in 14 years, going from 40.1% in 2005–2008 to 57.4% in 2016–2018 (*P* < 0.001); and (iv) although seven out of every ten nursing personnel are concentrated in metropolitan areas, the percentage of nurses in rural areas increased by 75% (from 4.8% in 2005–2008 to 8.4% in 2016–2018, *P* < 0.05) (Table 1).

**Table 1:** Main socio-demographic characteristics of nursing personnel in Mexico, 2005–2018

Period	From 2005 to 2006	From 2007 to 2009	From 2010 to 2012	From 2013 to 2015	From 2016 to 2018	*P* for trend
Weighted sample	795 012	1 312 910	1 408 368	1 680 496	1 926 978	
	Percentage and CI95%					
Women	93.5 [92.2 to 94.8]	92.7 [91.6 to 93.7]	90.1 [88.8 to 91.3]	87.1 [85.8 to 88.4]	86.0 [84.8 to 87.3]	*<* 0.001
Age (years)						
≤ 24	20.1 [17.7 to 22.4]	20.1 [18.3 to 22.0]	22.2 [20.3 to 24.2]	24.5 [22.8 to 26.3]	26.5 [24.8 to 28.3]	*<* 0.001
From 25 to 44	52.2 [49.3 to 55.1]	49.5 [47.2 to 51.8]	45.1 [42.8 to 47.4]	42.8 [40.8 to 44.8]	41.3 [39.4 to 43.3]	*<* 0.001
From 45 to 64	26.2 [23.6 to 28.8]	28.3 [26.3 to 30.2]	30.5 [28.2 to 32.7]	29.2 [27.4 to 31.1]	28.1 [26.4 to 29.8]	0.46
≥ 65	1.5 [0.8 to 2.2]	2.1 [1.3 to 2.9]	2.2 [1.6 to 2.8]	3.4 [2.5 to 4.3]	4.1 [3.2 to 5.0]	*<* 0.001
Marital status						
Married or union free	58.1 [55.2 to 61.0]	56.6 [54.4 to 58.9]	54.5 [52.2 to 56.7]	53.1 [51.0 to 55.1]	49.6 [47.7 to 51.6]	*<* 0.001
Single	31.8 [29.0 to 34.6]	31.4 [29.3 to 33.5]	34.8 [32.7 to 37.0]	36.7 [34.7 to 38.6]	39.1 [37.1 to 41.1]	*<* 0.001
Divorced or widowed	10.1 [8.4 to 11.7]	11.9 [10.5 to 13.4]	10.7 [9.4 to 12.0]	10.3 [9.1 to 11.4]	11.3 [10.0 to 12.5]	0.936
University-trained						
No	59.9 [57.2 to 62.5]	59.0 [56.8 to 61.3]	48.6 [46.3 to 50.9]	49.5 [47.4 to 51.6]	42.6 [40.5 to 44.7]	*<* 0.001
Yes	40.1 [37.5 to 42.8]	41.0 [38.7 to 43.2]	51.4 [49.1 to 53.7]	50.5 [48.4 to 52.6]	57.4 [55.3 to 59.5]	*<* 0.001
Residence place						
Rural	4.8 [3.5 to 6.1]	7.8 [6.3 to 9.4]	7.4 [6.0 to 8.7]	7.2 [5.8 to 8.6]	8.4 [7.1 to 9.6]	0.030
Semi-urban	12.4 [9.8 to 15.1]	10.7 [8.8 to 12.7]	11.5 [9.5 to 13.5]	11.4 [9.7 to 13.1]	12.7 [11.1 to 14.4]	0.478
Urban	16.9 [13.8 to 19.9]	17.2 [14.6 to 19.8]	15.9 [13.4 to 18.4]	18.0 [15.6 to 20.4]	17.0 [15.1 to 19.0]	0.789
Metropolitan	65.9 [62.3 to 69.4]	64.2 [61.2 to 67.2]	65.3 [62.4 to 68.2]	63.3 [60.7 to 66.0]	61.9 [59.6 to 64.2]	0.082

Estimations considered the design effect of the survey. Data source: National Occupation and Employment Survey (ENOE in Spanish) 2005–2018

The general labor profile of the nurses studied was constant throughout the 14 years analyzed, except the percentage that reported being economically active (decreasing from 66% in 2005–2006 to 60% in 2016–2018, *P* < 0.001), the percentage of nurses working in the health sector (decreasing from 72% in 2005–2006 to 65% in 2016–2018, *P* < 0.01), and the percentage of nurses working in a public institution (decreasing from 63% in 2005–2006 to 55% in 2016–2018, *P* < 0.01). In contrast, among the total of nurses, 95% reported being employed, 85% reported being subordinated and remunerated, and 94% reported having only one job throughout the 14 years (Table 2).

**Table 2:** Labor characteristics of nursing personnel in Mexico, 2005–2018

Period	From 2005 to 2006	From 2007 to 2009	From 2010 to 2012	From 2013 to 2015	From 2016 to 2018	*P* for trend
Weighted sample	795 012	1 312 910	1 408 368	1 680 496	1 926 978	
	Percentage and CI95%					
Economically active	65.6 [62.9 to 68.4]	66.3 [64.2 to 68.4]	64.1 [62.1 to 66.1]	63.0 [61.0 to 64.9]	60.4 [58.4 to 62.3]	*<* 0.001
Employed	95.3 [93.7 to 96.9]	96.7 [95.7 to 97.7]	95.6 [94.6 to 96.7]	94.7 [93.6 to 95.7]	95.2 [94.0 to 96.4]	0.158
Subordinated and remunerated	86.7 [84.2 to 89.3]	87.0 [85.1 to 88.9]	85.2 [83.4 to 87.0]	85.8 [84.1 to 87.5]	85.0 [83.2 to 86.7]	0.149
Having only one job	93.2 [91.6 to 94.9]	93.0 [91.7 to 94.3]	94.7 [93.6 to 95.7]	93.9 [92.9 to 95.0]	94.1 [93.1 to 95.2]	0.213
Having two or more jobs	6.8 [5.1 to 8.4]	7.0 [5.7 to 8.3]	5.3 [4.3 to 6.4]	6.1 [5.0 to 7.1]	5.9 [4.8 to 6.9]	0.213
Working in the health sector	72.0 [68.8 to 75.2]	70.3 [67.7 to 72.9]	69.3 [66.8 to 71.9]	69.0 [66.6 to 71.4]	65.3 [62.8 to 67.9]	0.001
Working in a public institution	62.9 [59.5 to 66.3]	62.5 [59.8 to 65.3]	62.1 [59.3 to 64.8]	60.5 [57.9 to 63.1]	54.6 [51.9 to 57.3]	*<* 0.001

Estimations considered the design effect of the survey. Data source: National Occupation and Employment Survey (ENOE in Spanish) 2005–2018

Four of the five indicators of precariousness increased among the nurses analyzed (Table 3): the percentage of people with no written contract (non-written agreement) increased by 8.6% (*P* < 0.10), the percentage of people with incomes lower than two times the minimum wage increased by 53.4% (*P* < 0.001), the percentage of nurses without social security increased by 41.8% (*P* < 0.001), and the percentage of those without a written contract, and of those without social benefits, grew by 40.7% (*P* < 0.01). Together, the percentage of this population that works under some condition of work precariousness increased notably during the years analyzed, increasing from 46.2% in 2005–2006 to 53.7% in 2018 (*P* < 0.001), with the middle and highest precariousness categories growing the most (12.3% and 60.8%, respectively, *P* < 0.05).

**Table 3:** Labor precariousness in nursing professionals in Mexico, 2005–2018

Period	From 2005 to 2006	From 2007 to 2009	From 2010 to 2012	From 2013 to 2015	From 2016 to 2018	*P* for trend
Weighted sample	795 012	1 312 910	1 408 368	1 680 496	1 926 978	
	Percentage and CI95%					
No written contract (non-written agreement)	15.5 [12.6 to 18.3]	13.1 [10.8 to 15.3]	12.2 [10.3 to 14.1]	15.6 [13.6 to 17.6]	16.8 [14.3 to 19.3]	0.063
Income lower than two times the minimum wage	16.1 [13.3 to 19.0]	16.4 [14.0 to 18.8]	17.8 [15.4 to 20.1]	20.4 [18.1 to 22.7]	24.7 [22.1 to 27.4]	*<* 0.001
With a partial or excessive workday	30.4 [27.2 to 33.7]	30.6 [27.8 to 33.4]	29.2 [26.5 to 32.0]	29.8 [27.3 to 32.2]	33.6 [30.9 to 36.3]	0.137
No social benefits	11.3 [9.0 to 13.6]	12.4 [10.4 to 14.4]	11.4 [9.6 to 13.3]	14.5 [12.6 to 16.4]	15.9 [13.5 to 18.2]	0.001
No social security	15.3 [12.6 to 17.9]	16.8 [14.5 to 19.2]	14.6 [12.6 to 16.5]	21.5 [19.2 to 23.9]	21.7 [19.1 to 24.2]	*<* 0.001
Work precariousness						
Non-precarious	53.8 [50.2 to 57.4]	53.3 [50.2 to 56.4]	55.3 [52.3 to 58.4]	50.7 [47.9 to 53.5]	46.3 [43.5 to 49.2]	*<* 0.001
Lowest	26.6 [23.4 to 29.8]	28.3 [25.5 to 31.0]	27.0 [24.3 to 29.7]	26.6 [24.1 to 29.0]	28.1 [25.5 to 30.7]	0.831
Middle	12.2 [9.6 to 14.8]	10.6 [8.7 to 12.4]	9.7 [8.0 to 11.3]	12.8 [11.0 to 14.6]	13.7 [11.8 to 15.6]	0.033
Highest	7.4 [5.5 to 9.3]	7.9 [6.2 to 9.6]	8.0 [6.3 to 9.7]	9.9 [8.3 to 11.6]	11.9 [9.7 to 14.1]	*<* 0.001

Estimations considered the design effect of the survey. Data source: National Occupation and Employment Survey (ENOE in Spanish) 2005–2018

**Fig. 2:**
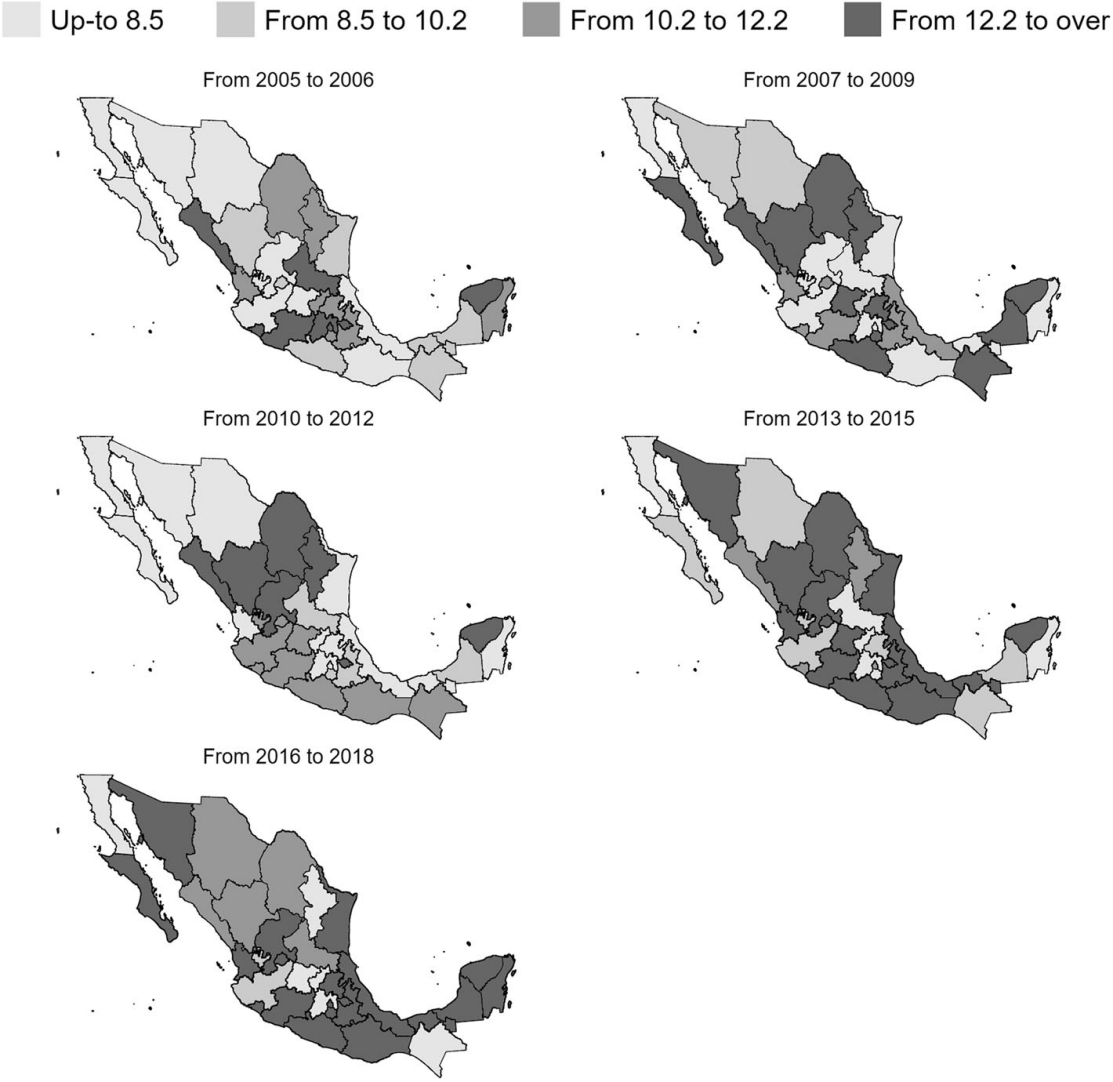
Geographical evolution of the middle and high labor precariousness in nursing professionals in Mexico, 2005–2006. Cutting thresholds based on the period from 2005 to 2006. Estimations considered the design effect of the survey. Data source: National Occupation and Employment Survey (ENOE in Spanish) 2005–2018

Finally, using the quartiles of the percentage of nurses working under conditions of medium or high precarization observed between 2005 and 2006 as a reference, Fig. 2 shows the generalized and important growth of the percentage of nurses working under these conditions in Mexico. Labor precarization has progressed from presenting important state heterogeneity between 2005 and 2006 to becoming much more geographically homogeneous between 2016 and 2018 (avg = 13.2%, C95% 11.6 to 14.8), which is a higher percentage than the state average observed during between 2005 and 2006 (10.1%, CI95% 8.7 to 11.6).

## Discussion

Nursing in Mexico is an occupation that has undergone important transformations in the last 15 years. The most obvious change is the increase in the level of training, moving from being an eminently technical occupation to one with university training, a shift that has been previously documented in the educational field [18]. Our results show that university-level nurses currently represent the highest proportion of all nurse-training categories in the labor market. In fact, they represent the highest percentage of all occupational categories in health, including medicine.

Despite the increase in nurse training levels, this study was able to document a notable and growing prevalence of work precariousness of each of its evaluated dimensions (economic, regulatory, and occupational safety) among Mexican nurses. In the 14-year period analyzed, the percentage of this population that worked under some precarious work condition increased by 18%, which represented almost six out of ten people. This finding matches recent analysis of various European countries, which suggests the existence of an increased risk of precarization for graduates of higher education institutions, with the risk being greater when educational institutions do not promote an early link to the labor market through a practice-oriented training [19]. In Mexico, plans for nurse training are heterogeneous in terms of their theoretical/practical orientation, which could influence employers’ decisions at the time graduates are inserted into the labor market.

An underlying approach to our analysis is that precarization is multidimensional. When we break down the global indicator of precariousness, we find a notable growth of the percentage (54%) of people with incomes lower than two times the minimum wage (282 USD per month), the percentage of people with no written contract (8%), and the percentage without social security (42%). In his analysis of public workers in Argentina, Diana-Menéndez [20] argued that precarization cannot be understood as a dichotomous problem, but rather, we need to follow a multidimensional approach to understand how precarization can have differential effects on the labor force.

In line with previous studies, our findings also point to an incipient process of masculinization [21–23], accompanied by an age restructuring, with the group of highest growth being those 24 or younger (32%). These two aspects may be producing differential patterns on the precarization of nurses as it has been documented that male and middle-age nurses enjoy different labor market conditions than women [24, 25]. In addition, one third of Mexican nurses do not work in the health sector, which reveals the presence of underemployment and confirms a regulatory failure in the nursing labor market, as it implies the search for opportunities in non-health market areas where the competencies obtained in nurse training will not be at stake. On the other hand, in the years analyzed, 85% of the nurses declared being salaried. It was also observed that the participation of nurses in the public sector oscillated during this period but trended towards reduced participation. The involvement in public health institutions during this time period has been based on the hiring of health personnel, including nurses, in precarious conditions [26] as well as by the increased demand for nurses to cover internships in public and social security health units.

Our results also revealed that the generalized growth of the percentage of nurses working under conditions of medium or high precarization across all federal states in México has gone from percentages around 10.1% during the years 2005–2006 to be practically homogeneous geographically in the period 2016–2018 (avg = 13.2%). Data throughout this period may represent a transitional phase marking a before and after in the phenomenon of precarization. Most probably, the advancement of precarization is related to specific contracting policies that started by mid-1980s as part of a structural adjustment strategy. The government decided to stop creating permanent positions in public units. Until the end of 1990s, the number of jobs in the Ministry of Health and other public health institutions remained stagnant and new recruits only filled the positions of the retired or the dead. Short-term contracting also expanded to cover temporary absentees. It was not until 2003 when Seguro Popular was created that the Ministry of Health was able to offer full-time positions but under precarious conditions [4, 5]. These types of contracts were offered in all states following the advancement of Seguro Popular that, between 2003 and 2007, progressively financed all States Ministry of Health services. By the beginning of its administration, the new government announced an austerity policy, meaning the possibility of cutting thousands of temporary contracts for nurses across the country. It this policy comes into fruition various unintended consequences of this policy could emerge [27, 28]. We may see a reduction in the number of precarious jobs in public institutions. But also a reduction of nurses in primary care centers and rural areas. We may also see an increase of unemployment and underemployment among nurses and a shift of nurses to private market areas where precarious work is more widespread.

This study has limitations that are common to all observational studies. Two in particular stand out: (i) the relationships identified should be considered as statistical associations and not as causal relationships and (ii) it was not possible to consider the condition of precariousness analyzed or the time performing the nursing activity in the analysis.

In addition, we do not fail to recognize that the very definition of job precariousness also presents some limitations: first, our construct of precariousness assigns the same weight to each of its dimensions. Although it was beyond the purpose of our research, it would be desirable to have an explicit exercise (i.e., a meta-analysis or a consultation with experts, etc.) to define these weights based on their importance in labor market participation. The second and main limitation is the impossibility of including people who, in spite of being nurses, do not fulfill the conditions of being economically active, employed, and subordinated and remunerated, and that in the 14 years analyzed represented 45% of the population of interest. Although part of this group does not work by personal decision, if there is a more comprehensive and inclusive definition (which allows for example, being unemployed), surely the prevalence of precarious work would be much higher. Also, future literature should focus on the use of metrics that more closely approximate the precariousness of work and the weight of its dimensions, particularly in low- and middle-income economies.

The nursing labor market in Mexico is in an acute phase of transformation, and precarization has become an indicative element of its current conditions. Analysts of nursing labor markets in other countries [29] have warned that the outsourcing of hiring has introduced risks that did not exist when hiring was carried out directly between the worker and the employing institution. According to this approach, outsourcing hiring as a mechanism for seeking flexible employment relations that permit institutions to reduce or increase their workforce is an important determinant of employment precariousness [30, 31]. Flexible contracting has affected all areas of the economy, including health. Since the structural reforms of the 1990s, market logic has imposed itself on public institution bureaucratic logic.

In Mexico, the health system was not formally privatized, but at present, there is ample private participation both in the supply of hospital services and in the hiring of health personnel [32, 33]. The participation of companies that outsource employment has increased in recent years. These companies take the responsibility of hiring the workers, for a payment, on behalf of the public institutions that transfer the risk of labor liability. Even in cases where public institutions hire, they do so on the basis of contracts with defined temporality and without legal benefits, but outsourcing is displacing this modality [34].

The implications of this article are potentially relevant for other low- and middle-income countries. First, it is important to note that the precarization of nurses’ jobs is a complex phenomenon related to multiple factors that have been documented quantitatively. Among these factors are a wide variety of global and national elements. It is possible to identify common and distinct elements for different countries. Precarization is a growing phenomenon that accompanies structural changes, the role of the government, and the capacity of health systems as mass employers of health personnel. In Latin America, a common element is that most health systems in countries of this region underwent reforms that incorporated market elements in different modalities. The reforms have been associated to the deterioration of the contractual link between workers and institutions, whose role has been reduced to driving the system by transferring the provision of services to other actors to a large extent. These changes have had effects particularly in highly salaried occupations, nursing being one of them.

The implications of precarization in relation to the condition of individuals and the labor markets into which they work are diverse [35]. In most Latin American countries, nurses are the main human resource, which is why they are a fundamental component in achieving the objectives of health systems. They are responsible for an increasing volume of provision of health services with direct implications for the achievement of universal health coverage (UHC) [36]. Precarization could represent an obstacle in allowing highly trained nurses to engage the labor market in favorable conditions and reduce their capacity to be a health systems asset in achieving effective health coverage. This condition tends to detach the traditional link between workers and employers, not only that of nurses, generating a loss of service capacity and a reduction in productivity and quality.

## Conclusions

Considered as a global phenomenon, precarization poses an enormous challenge for national governments. This is particularly important for countries that seek to be competitive in international markets by maintaining tight control of the cost of their workforce, which often means skimping on labor rights. However, for health systems that do not seek to compete in global markets, but rather to offer better health services to their beneficiary populations, precarization is a high-risk phenomenon due to the potential loss of quality of services provided. Consequently, it is the responsibility of governments to regulate the production of nurses based on models that adjust for health institution demand in the medium and long term, to establish working conditions that protect workers beginning with granting international labor rights in every country, and to protect the workforce in an occupation such as nursing, where the majority of the members are women.

## Data Availability

The dataset used and/or analyzed during the current study are available from the corresponding author on request.
